# Importance of Timing of Platelet Lysate-Supplementation in Expanding or Redifferentiating Human Chondrocytes for Chondrogenesis

**DOI:** 10.3389/fbioe.2020.00804

**Published:** 2020-07-08

**Authors:** Margot Rikkers, Riccardo Levato, Jos Malda, Luciënne A. Vonk

**Affiliations:** ^1^Department of Orthopaedics, University Medical Center Utrecht, Utrecht University, Utrecht, Netherlands; ^2^Department of Equine Sciences, Faculty of Veterinary Medicine, Utrecht University, Utrecht, Netherlands

**Keywords:** platelet lysate, chondrocyte, cartilage regeneration, ACI, xeno-free

## Abstract

Osteoarthritis (OA) in articular joints is a prevalent disease. With increasing life expectancy, the need for therapies other than knee replacement arises. The intrinsic repair capacity of cartilage is limited, therefore alternative strategies for cartilage regeneration are being explored. The purpose of this study is first to investigate the potential of platelet lysate (PL) as a xeno-free alternative in expansion of human OA chondrocytes for cell therapy, and second to assess the effects of PL on redifferentiation of expanded chondrocytes in 3D pellet cultures. Chondrocytes were isolated from human OA cartilage and subjected to PL in monolayer culture. Cell proliferation, morphology, and expression of chondrogenic genes were assessed. Next, PL-expanded chondrocytes were cultured in 3D cell pellets and cartilage matrix production was assessed after 28 days. In addition, the supplementation of PL to redifferentiation medium for the culture of expanded chondrocytes in 3D pellets was evaluated. Glycosaminoglycan (GAG) and collagen production were evaluated by quantitative biochemical analyses, as well as by (immuno)histochemistry. A dose-dependent effect of PL on chondrocyte proliferation was found, but expression of chondrogenic markers was decreased when compared to FBS-expanded cells. After 28 days of subsequent 3D pellet culture, GAG production was significantly higher in pellets consisting of chondrocytes expanded with PL compared to controls. However, when used to supplement redifferentiation medium for chondrocyte pellets, PL significantly decreased the production of GAGs and collagen. In conclusion, chondrocyte proliferation is stimulated by PL and cartilage production in subsequent 3D culture is maintained. Furthermore, the presences of PL during redifferentiation of 3D chondrocyte strongly inhibits GAG and collagen content. The data presented in the current study indicate that while the use of PL for expansion in cartilage cell therapies is possibly beneficial, intra-articular injection of the product in the treatment of OA might be questioned.

## Introduction

Focal cartilage defects occur frequently in young and active patients and cause serious limitations on both joint function and the patient’s mobility and quality of life (Heir et al., 2010). As cartilage is an avascular tissue, spontaneous healing of the tissue is limited. In addition, with increasing life expectancy, the patient population with osteoarthritis (OA) is growing continuously (Cross et al., 2014).

A well-established surgical procedure to treat cartilage defects is autologous chondrocyte implantation (ACI). Long-term follow-up studies have demonstrated hyaline-like cartilage formation and satisfactory clinical outcome up to 20 years post-surgery (Brittberg, 2008; Peterson et al., 2010). Nevertheless, a major part of treated patients shows signs of fibrocartilage formation in the regenerated area (McCarthy et al., 2018). As fibrocartilage is mechanically inferior to hyaline cartilage, it is unfit to fulfill the natural functions of hyaline cartilage and therefore more prone to degradation (Messner and Maletius, 1996; Knutsen et al., 2004). One of the causes of the fibrocartilage formation is dedifferentiation of chondrocytes during the *in vitro* expansion phase (Schnabel et al., 2002), which is a requirement to obtain a sufficient amount of cells for autologous cell transplantation. Maintaining chondrogenic redifferentiation capacity of chondrocytes during expansion is essential for improving the quality of the regenerated cartilage and thus potentially improves clinical outcome.

Platelet-rich plasma (PRP) is a blood product containing high growth factor levels that has been used for various applications over the past decades (Sánchez et al., 2007; Mei-Dan et al., 2012; Smith, 2015; Zhang et al., 2018). While variations in content and production methods exist, PRP consistently contains a high concentration of blood platelets. In orthopedics, PRP and PRP-derivates like platelet lysate (PL) can be used for applications such as intra-articular injection for the treatment of knee osteoarthritis (Filardo et al., 2015b). Moreover, as it is a rich source of growth factors, human PL also shows potential to be used in cell culture as a xeno-free alternative to bovine serum, possibly as a pooled off-the-shelf media supplement. In clinical cell therapy, PL is already used for the expansion of cells (de Windt et al., 2016).

The effect of *in vitro* expansion in the presence of PL on the chondrogenic potential of chondrocytes remains unclear. While most studies agree that PRP and PL have a stimulatory effect on chondrocyte proliferation (Drengk et al., 2008; Pereira et al., 2013), contradictory results have been reported on anabolic effects of PRP-derivates on cartilage matrix formation by chondrocytes (Drengk et al., 2008; Pereira et al., 2013; Xie et al., 2014).

Therefore, the current study aims to investigate the effect of PL on the chondrogenic potential of chondrocytes. More specifically, this study looked into the effect of PL on chondrocytes during expansion and subsequent 3D culture, as well as effects on matrix production in 3D cultures while being exposed to PL.

## Materials and Methods

### Experimental Design and Study Outline

To test the hypothesis whether PL will maintain chondrogenic capacity of culture expanded chondrocytes, chondrocyte monolayers were subjected to various concentrations of PL and compared to culture in fetal bovine serum (FBS). To subsequently assess cartilage-like matrix formation, chondrocytes were harvested and cultured in 3D cell pellets. The production of sulfated GAGs and collagens was measured biochemically, as well as histologically and by gene expression to compare between groups. In addition, the hypothesis that PL will improve redifferentiation of chondrocytes was tested using a similar 3D cell pellet culture system where PL was added fresh into the redifferentiation medium each medium change. Production of GAGs and collagens was assessed similarly as above.

### Platelet Lysate Preparation and Growth Factor Quantification

Platelet lysate was prepared from human blood collected in 3.2% sodium citrate-containing tubes. Blood was obtained through the Mini Donor Service, a blood donation facility for research purposes approved by the medical ethics committee of the University Medical Center Utrecht, Netherlands. All donors have provided written informed consent, in accordance with the declaration of Helsinki. All donors were reported being healthy and free from antiplatelet drugs or non-steroid anti-inflammatory drugs. Platelet-rich plasma was prepared by centrifugation of whole blood at 130 *g* for 15 min, upon which pelleted erythrocytes were discarded and the plasma layer was concentrated by a second centrifugation cycle at 250 *g* for 15 min. Platelet count was measured using a CELL-42 DYN Emerald Hematology Analyzer (Abbott, United States). Platelets were activated to release their growth factors by three freeze-thaw cycles, after which samples were centrifuged at 8,000 *g* for 10 min and supernatant PL was stored at −20°C until use. Pooled PL from at least three donors was used in each experiment. The concentrations of human growth factors PDGF-AB, TGF-β1, VEGF, and FGF-2 were quantitatively determined using enzyme-linked immunosorbent assays (ELISA) according to the manufacturer’s instructions (DuoSet ELISA kits, R&D Systems, Minneapolis, MN, United States).

### Donors and Cell Isolation

Osteoarthritic cartilage was obtained from redundant material from patients who had undergone total knee arthroplasty. The material is collected anonymously according to the Medical Ethics regulations of the University Medical Center Utrecht and approved by the local medical ethics committee (University Medical Center Utrecht). Cartilage was dissected from the underlying bone, rinsed in phosphate-buffered saline (PBS) and cut into 2 mm pieces. The pieces were digested in 0.2% (w/v) pronase (Sigma-Aldrich) in Dulbecco’s modified Eagle’s medium (DMEM, 31966; Gibco, Netherlands) with 1% penicillin/streptomycin (100 U/mL, 100 mg/mL; Gibco) at 37°C for 2 h, followed by overnight digestion in 0.075% (w/v) collagenase type II (CLS-2, Worthington, Lakewood, NJ, United States) in DMEM supplemented with 10% (v/v) heat-inactivated FBS (Biowest) and 1% penicillin/streptomycin under agitation. The cells were then filtered through a 70-μm cell strainer (Greiner Bio-One), washed, and counted. Chondrocytes were expanded using Expansion medium (DMEM supplemented with 1% penicillin/streptomycin and 10% FBS). Cells were cryopreserved in liquid nitrogen until use.

### Cell Culture

Passage 1 chondrocytes (*n* = 6 donors, age 61–75, average 69 years) were seeded in 24-well plates at 1,500 cells/cm^2^ and cultured for 7 days in Expansion medium, where FBS was replaced with 0.01, 0.1, 1, 2, or 5% PL (all v/v). All media containing PL were supplemented with 3.3 U/mL heparin (Sigma-Aldrich) to prevent coagulation. Control conditions were cultured in Expansion medium with 10% FBS, with and without heparin. As a negative control, chondrocytes were expanded with serum-free medium (DMEM supplemented with 2% human serum albumin (HSA; Sanquin Blood Supply Foundation), 2% insulin-transferrin-selenium-ethanolamine (ITS-X; Gibco), 0.2 mM L-ascorbic acid 2-phosphate (ASAP; Sigma-Aldrich), and 1% penicillin/streptomycin). This did not lead to a sufficient number of chondrocytes to be used for further pellet culture and therefore this condition was not included in the following experiments. Photomicrographs can be found in Supplementary Figure S1.

For the 3D pellet culture, chondrocytes (*n* = 5 donors, age 55–75, average 65 years) were expanded in monolayers in Expansion medium with either 10% FBS, 1, or 5% PL and heparin up to passage 2. Following expansion, pellets containing 2.5 × 10^5^ cells were formed in ultra-low attachment 96-well round-bottom plate wells (Corning) by centrifugation at 300 *g* for 5 min. Pellets were cultured for 28 days in Redifferentiation medium (DMEM supplemented with 2% HSA, 2% ITS-X, 0.2 ASAP, and 1% penicillin/streptomycin).

For evaluating the effects of PL on chondrogenic redifferentiation, chondrocytes (*n* = 6 donors, age 54–84, average 65 years) were first expanded in Expansion medium with 10% FBS. Pellets were then cultured for 28 days in Redifferentiation medium either or not supplemented with 1 or 5% PL and heparin.

### Immunofluorescent Staining

Expanded monolayers were fixed after 7 days with 10% buffered formalin. Fixed monolayers were permeabilized using 0.2% Triton X-100 (Sigma-Aldrich) in PBS for 20 min, followed by incubation with phalloidin (TRITC-conjugated; 1/200 dilution in PBS; Sigma-Aldrich) for 1 h. Nuclei were stained with 100 ng/mL 4’,6-diamidino-2-phenylindole (DAPI; Sigma-Aldrich) in PBS for 5 min. Monolayers were imaged using an upright fluorescent microscope (BX51; Olympus).

### Biochemical Analysis of Pellets

Pellets were harvested after 28 days to measure glycosaminoglycan (GAG), collagen, and DNA content. Samples were digested overnight in a papain digestion buffer (250 μg/mL papain; Sigma-Aldrich, 0.2 M NaH_2_PO_4_, 0.1M EDTA, 0.01M cysteine, pH 6) at 60°C.

Sulfated GAG content was quantified using a dimethylmethylene blue (DMMB; pH 3) assay. The 525/595 nm absorbance ratio was measured using chondroitin-6-sulfate (Sigma-Aldrich) as a standard.

Total collagen content was calculated from the hydroxyproline content (Neuman and Logan, 1950). Papain digests were lyophilized and hydrolyzed in 4 M NaOH (Sigma-Aldrich) in Milli-Q water overnight at 108°C, after which samples were neutralized with 1.4 M citric acid (Sigma-Aldrich) in Milli-Q water. 50 mM freshly prepared Chloramin-T (Merck) in oxidation buffer was added and incubated for 20 min under agitation. Subsequently, 1.1 M freshly prepared dimethylaminobenzoaldehyde (Merck) in 25% (v/v) perchloric acid (Merck) in 2-propanol (Sigma-Aldrich) was added and incubated for 20 min at 60°C. Samples were cooled and absorbance read at 570 nm using hydroxyproline (Merck) as a standard.

Total DNA content was quantified using a Quant-iT PicoGreen dsDNA assay (Invitrogen) according to the manufacturer’s instructions. Fluorescence was measured at 485 nm excitation and 535 nm emission by a microplate fluorometer (Fluoroskan Ascent).

### Histology

Pellets were processed for histology by fixation in 10% buffered formalin, dehydration through graded ethanol steps, clearing in xylene, and subsequent embedding in paraffin. 5 μm sections were cut and deparaffinised before staining. Sections were stained for GAGs with 0.125% safranin-O (Merck) counterstained with 0.4% fast green (Sigma-Aldrich) and Weigert’s hematoxylin (Clin-Tech). Collagen deposition was evaluated by immunohistochemistry, with appropriate primary antibodies for type II collagen (II-II6B3; DHSB; 1:100 in PBS/BSA 5%) and type I collagen (EPR7785, BioConnect; 1:400 in PBS/BSA 5%). Samples were blocked using 0.3% H_2_O_2_, followed by antigen retrieval with pronase (1 mg/mL; Sigma-Aldrich) for 30 min at 37°C and hyaluronidase (10 mg/mL; Sigma-Aldrich) for 30 min at 37°C. Next, sections were blocked using bovine serum albumin (BSA; 5% (w/v) in PBS) for 30 min, followed by overnight incubation with the primary antibody at 4°C. After washing, type II collagen sections were incubated with a goat-anti-mouse IgG HRP-conjugated (DAKO, P0447; 1:100 in PBS/BSA 5%) and type I collagen sections with Envision + System-HRP anti-rabbit (DAKO, K4003), both for 1 h at room temperature. Next, both stainings were developed using 3,3’-diaminobenzidine (DAB, Sigma-Aldrich). Sections were counterstained with Mayer’s hematoxylin (Klinipath) and mounted in DPX mounting medium (Merck).

### Real-Time PCR

Total RNA was isolated from monolayers and pellets using TRIzol (Invitrogen) according to the manufacturer’s instructions. Total RNA (200–500 ng) was reverse transcribed using the High-Capacity cDNA Reverse Transcription Kit (Applied Biosystems). Real-time polymerase chain reactions (PCRs) were performed using iTaq Universal SYBR Green Supermix (Bio-Rad) in a LightCycler 96 (Roche Diagnostics) according to the manufacturer’s instructions. The amplified PCR fragments extended over at least one exon border (except for 18S). Relative gene expression was calculated using 18S as a housekeeping gene. Primers (Invitrogen) used for real-time PCR are listed in Table 1.

**TABLE 1:** Primer sequences used for real-time PCR.

Target gene	Oligonucleotide sequence (5’ to 3’)	Annealing temperature (°C)	Product size (bp)
*18S*	Fw: GTAACCCGTTGAACCCCATT Rv: CCATCCAATCGGTAGTAGCG	57	151
*COL2A1*	Fw: AGGGCCAGGATGTCCGGCA Rv: GGGTCCCAGGTTCTCCATCT	57	195
*ACAN*	Fw: CAACTACCCGGCCATCC Rv: GATGGCTCTGTAATGGAACAC	56	160
*COL1A1*	Fw: TCCAACGAGATCGAGATCC Rv: AAGCCGAATTCCTGGTCT	57	191
*SOX9*	Fw: CCCAACGCCATCTTCAAGG Rv: CTGCTCAGCTCGCCGATGT	60	242
*ACTA2*	Fw: ATGCCATCATGCGTCTGGAT Rv: ACGCTCAGCAGTAGTAACGA	60	101
*NOTCH1*	Fw: AAGCTGCATCCAGAGGCAAAC Rv: TGGCATACACACTCCGAGAACAC	60	172
*CDH2*	Fw: GCGTCTGTAGAGGCTTCTGG Rv: GCCACTTGCCACTTTTCCTG	60	293

*Forward (Fw) and reverse (Rev) primers. COL2A1, collagen type II alpha 1 chain; ACAN, aggrecan; COL1A1, collagen type I alpha 1 chain; SOX9, SRY-box transcription factor 9; ACTA2, actin alpha 2, smooth muscle; NOTCH1, notch receptor 1; CDH2, cadherin 2.*

### Statistical Analysis

Each experiment was performed with cells from six donors. For the monolayer expansion, three wells were used for each condition. For pellet cultures, three pellets were used for biochemical analyses, three for gene expression analysis, and two for histology. Data are expressed as mean ± standard deviation (SD). Data were statistically analyzed using the GraphPad Prism 7.0 software package (GraphPad Software, United States). Normal distribution of the data was checked with a Shapiro-Wilk test (*p* > 0.05) and homogeneity with a Levene’s test. When the data was normally distributed and samples were homogeneous, a one-way analysis of variance (ANOVA) was performed with Dunnett’s *post hoc* test. When the data was non-normally distributed, a Kruskal-Wallis test was performed with Dunn’s *post hoc*. A value of *p* < 0.05 was considered statistically significant.

## Results

### Platelet Lysate Characterization

To characterize the PL product, cell and platelet concentrations were counted and growth factor concentrations were determined in six batches of PL by ELISA (Table 2). Manually prepared PL was deprived from erythrocytes (19 ± 4 × 10^9^/L) and leukocytes (0.3 ± 0.1 × 10^9^/L), while platelet concentrations were maintained (458 ± 87 × 10^9^/L). Furthermore, we found high concentrations of PDGF-AB (36142 ± 2518 pg/mL) and TGF-β1 (65379 ± 3889 pg/mL), while concentrations of FGF-2 (49 ± 10 pg/mL) and VEGF (150 ± 53 pg/mL) were less.

**TABLE 2:** Cell and growth factor concentrations in platelet lysate.

**Content (mean ± SEM)**	
Erythrocytes (10^9^/L)	19 ± 4
Leukocytes (10^9^/L)	0.3 ± 0.1
Platelets (10^9^/L)	458 ± 87
PDGF-AB (pg/mL)	36,142 ± 2,518
TGF-β1 (pg/mL)	65,379 ± 3,889
FGF-2 (pg/mL)	49 ± 10
VEGF (pg/mL)	150 ± 53

*Cell concentrations in PL were determined using a CELL-42 DYN Emerald Hematology Analyzer. Growth factor concentrations in PL were quantified by enzyme-linked immunosorbent assays (ELISA). All values are expressed as mean ± SEM (n = 6).*

### Chondrocyte Proliferation Is Stimulated in the Presence of Platelet Lysate During Expansion

**FIGURE 1:**
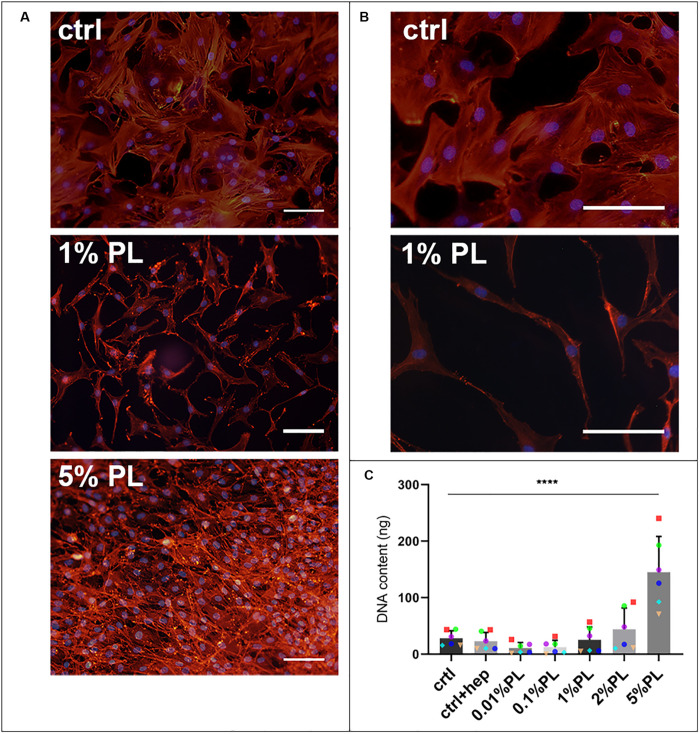
Effect of platelet lysate on chondrocyte proliferation. Phalloidin/DAPI staining of passage 1 chondrocytes after 7 days in monolayer culture in expansion medium supplemented with 10% FBS, 1, and 5% PL **(A)**. Higher magnification of the photomicrographs showing the morphology of chondrocytes expanded with 10% FBS and 1% PL **(B)**. DNA content of the monolayer conditions **(C)**. All scale bars = 100 μm. Data are presented as mean ± SD. ****p < 0.0001.

Supplementation of Expansion medium with PL enhanced chondrocyte proliferation in a dose-dependent manner. Immunocytochemical staining of monolayers with phalloidin suggested an increase in cell amount after 7 days of exposure to PL (Figure 1A). These findings were confirmed by quantification of DNA content (Figure 1C). Furthermore, changes in cellular morphology were observed. Chondrocytes cultured in the presence of PL exhibited an elongated shape opposed to a more spindle-like shape for cells in control expansion cultures (Figure 1B).

**FIGURE 2:**
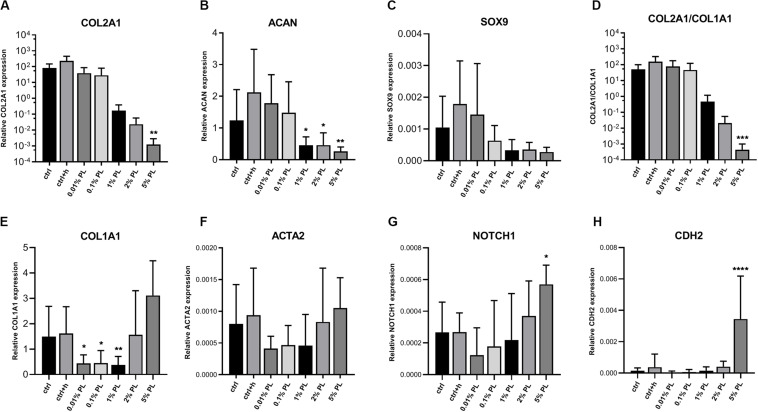
Gene expression of platelet lysate-expanded chondrocytes in monolayers. Gene expression type II collagen (COL2A1, **A**), aggrecan (ACAN, **B**), SRY-box transcription factor 9 (SOX9, **C**), ratio of type II/type I collagen (COL2A1/COL1A1, **D**), type I collagen (COL1A1, **E**), actin alpha 2, smooth muscle (ACTA2, **F**), notch receptor 1 (NOTCH1, **G**) and cadherin 2 (CDH2, **H**) by chondrocytes in monolayer culture in expansion medium supplemented with 10% FBS (ctrl), 10% FBS and 3.3 U/mL heparin (ctrl + h), 0.01% platelet lysate (PL), 0.1, 1, 2, and 5% PL (all PL conditions contain heparin), normalized for the housekeeping gene 18S. Data are shown as mean ± SD. *p < 0.05; **p < 0.005; ***p < 0.0005; ****p < 0.0001.

In contrast to the dose-dependent increase in DNA content, expression of chondrogenic genes coding for type II collagen (COL2A1), aggrecan (ACAN), and SRY-box transcription factor 9 (SOX9) decreased in a dose-dependent manner compared to the control group (Figures 2A–D). Expression of dedifferentiation marker type I collagen (COL1A1) was increased with increasing PL concentrations, while fibroblast marker actin alpha 2, smooth muscle (ACTA2) did not show significant changes (Figures 2E,F). Notch receptor 1 (NOTCH1) and cadherin 2 (CDH2), genes associated with stemness, were significantly increased when chondrocytes were exposed to culture medium containing 5% PL (Figures 2G,H).

### Chondrocytes Expanded in the Presence of Platelet Lysate Maintain Their Redifferentiation Potential

**FIGURE 3:**
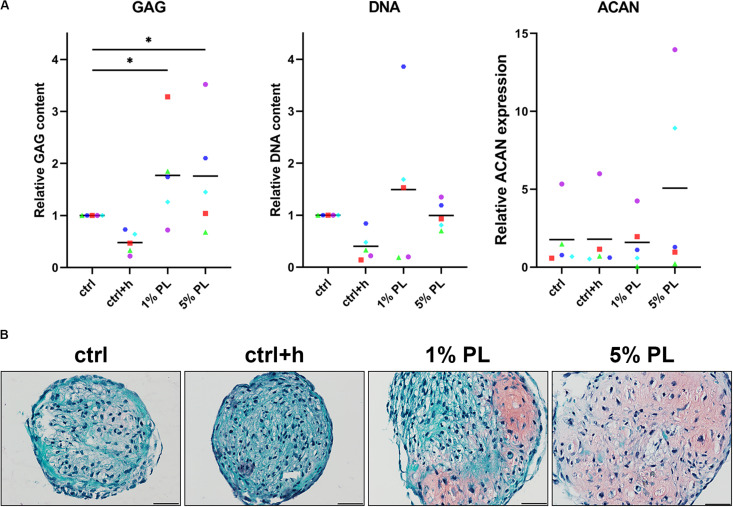
Glycosaminoglycan production of chondrocytes in 3D pellet cultures. Glycosaminoglycan (GAG) deposition and aggrecan (ACAN) gene expression in pellet cultures of passage 2 chondrocytes expanded in medium supplemented with 10% FBS (ctrl) with and without heparin (+h), 1- and 5% human platelet lysate (PL) cultured for 28 days in redifferentiation medium. **(A)** Biochemical analysis of GAGs and DNA content and relative expression of ACAN (normalized against the housekeeping gene 18S). Data are presented per donor and overall mean. *p < 0.05. **(B)** Histological assessment by safranin-O. Scale bar = 100 μm.

To compare chondrogenic potential of PL-expanded chondrocytes and chondrocytes expanded in the presence of FBS, 3D pellet cultures were performed to allow for neo-cartilage extracellular matrix (ECM) formation. Pellets consisting of PL-expanded chondrocytes were found to produce significantly more GAGs after 28 days when compared to the control conditions (Figure 3A). A slight qualitative increase in GAG production was seen across all chondrocyte donors used in this experiment (Figure 3B and Supplementary Figure S2). However, pellets consisting of 5% PL-expanded chondrocytes did not evidently show an improvement in GAG production on histology when compared to 1% PL-expanded chondrocyte pellets. Additionally, PL-expanded chondrocyte pellets did not present an increase in ACAN gene expression (Figure 3A).

**FIGURE 4:**
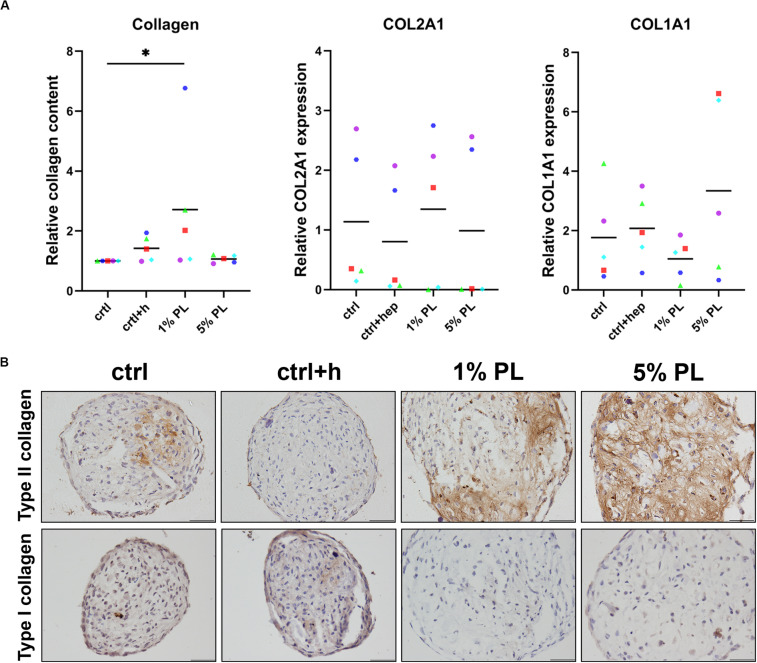
Collagen production of chondrocytes in 3D pellet cultures. Collagen deposition and type II (COL2A1) and I collagen (COL1A1) gene expression in pellet cultures of passage 2 chondrocytes expanded in medium supplemented with 10% FBS (ctrl) with and without heparin (+h), 1- and 5% human platelet lysate (PL) cultured for 28 days in redifferentiation medium. **(A)** Biochemical analysis of total collagen content and relative expression of COL2A1 and COL1A1 (normalized against the housekeeping gene 18S). Data are presented per donor and overall mean. *p < 0.05. **(B)** Histological assessment by type II and I collagen immunohistochemistry. Scale bar = 100 μm.

To further investigate the chondrogenic potential of PL-expanded chondrocytes, production of collagen was determined. Quantitative biochemical analysis of total collagen content revealed an increase in 1% PL-expanded chondrocyte pellets compared to the control conditions. However, no differences were found on gene expression level of COL2A1 and COL1A1 (Figure 4A). Immunohistochemical staining revealed a slight increase in type II collagen production in two out of five donors. Overall, no evident increase of type II collagen was seen in experimental groups, in line with the mRNA data (Figure 4B and Supplementary Figure S2). Furthermore, staining for type I collagen was absent in all conditions (Figure 4B).

Taken together, these data suggest that the chondrogenic differentiation capacity is preserved in osteoarthritic chondrocytes upon expansion with PL.

### Platelet Lysate Strongly Inhibits Matrix Production During Redifferentiation of Chondrocytes

To test whether PL has a chondrogenic effect on osteoarthritic chondrocytes *in vitro*, chondrocytes were cultured in 3D pellets, after which PL was added to the Redifferentiation medium. For these experiments, all cells were expanded using conventional Expansion medium containing 10% FBS, instead of PL.

**FIGURE 5:**
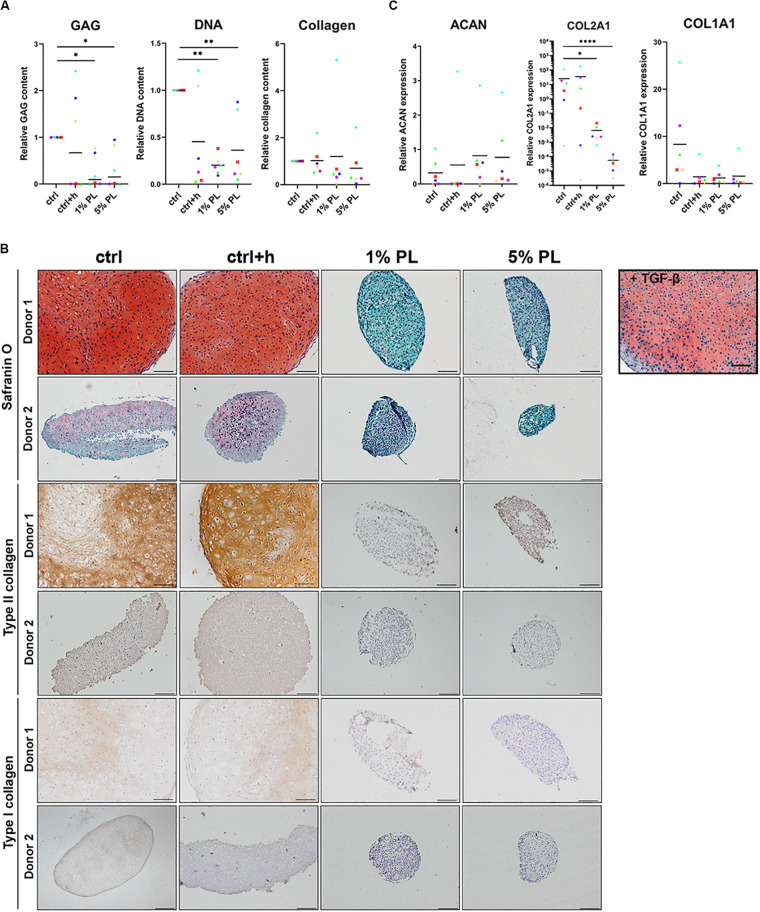
Matrix production and gene expression of chondrocytes in 3D pellet cultures supplemented with platelet lysate. **(A)** Glycosaminoglycan (GAG), collagen, and DNA content after 28 days of culture in redifferentiation medium (ctrl) with and without heparin (+h) and in the presence of 1- and 5% human platelet lysate (PL). **(B)** Cartilage production for one donor with high chondrogenic potential (Donor 1) and one donor with low chondrogenic potential (Donor 2) as determined by histological staining of GAGs by safranin-O, and immunohistochemical staining for type II and I collagen. Scale bar = 100 μm. **(C)** Relative gene expression of type II collagen (COL2A1), aggrecan (ACAN), and type I collagen (COL1A1), normalized for the housekeeping gene 18S. Data are presented per donor and overall mean. *p < 0.05; **p < 0.005; ****p < 0.0001.

Biochemical analyses revealed a significant decrease in GAG production in pellets differentiated in the presence of 1 or 5% PL. Further, cell numbers also decreased as confirmed by DNA quantification analysis. Quantitative analysis for total collagen did not reveal any difference between experimental and control conditions (Figure 5A). For donors with high chondrogenic capacity (Figure 5B, Donor 1) as well as donors having low chondrogenic capacity (Figure 5B, Donor 2), addition of PL during redifferentiation strongly inhibited GAG, type II and type I collagen formation. Histological stainings for GAGs and type II collagen of all donors used in this experiment are displayed in Supplementary Figure S3, confirming the uniform effect of PL on inhibition of cartilage matrix formation. Expression of chondrogenic marker COL2A1 significantly decreased in pellets cultured in the presence of 1 and 5% PL, while no difference was found in the expression of ACAN and COL1A1 (Figure 5C).

These data reveal a considerable inhibition of cartilage matrix production by PL in chondrocyte pellets, independent of chondrogenic capacity of the donor.

## Discussion

The dedifferentiation of chondrocytes during expansion is a major challenge in the successful application of ACI as this causes the formation of fibrocartilage. PRP and PL provide a rich source of growth factors, which can provide an autologous supplement for *in vitro* cell culture. While some studies report on the stimulation of chondrocyte proliferation, chondrogenesis, and diminishing catabolic effects in the treatment of OA, consensus on this topic does not exist. Moreover, very limited work has been done on the capacity of PL to rescue cartilage matrix production in cultured OA chondrocytes.

The aim of this study was to elucidate the effects of PL on OA chondrocytes *in vitro*. We confirmed that PL exerts a stimulatory effect on the proliferation of chondrocytes. A dose-dependent effect on chondrocyte proliferation by PL was found, as determined by the quantification of DNA in the monolayer samples. This result is in line with previous studies where PL was compared to FBS-containing expansion medium (Choi et al., 1980; Gaissmaier et al., 2005; Pereira et al., 2013; Hildner et al., 2015). In addition to the stimulation of proliferation, we found a decreased expression of chondrogenic marker genes COL2A1 and ACAN, and an increase of the dedifferentiation marker COL1A1 compared to FBS-expanded chondrocytes. Taken together, this suggests an increased dedifferentiation of chondrocytes during expansion in PL-supplemented medium. Interestingly, a recent study reports on increased expression of chondrogenic markers and decreased expression of dedifferentiation markers in MSCs and cartilage progenitor cells expanded using different concentrations of PL and the same effect was to a lesser extent visible in chondrocytes (Wang et al., 2019). Additionally, we found that chondrocytes expanded with the highest concentration of PL exhibited an increased expression of CDH2 and NOTCH1. CDH2 has been described to be involved in embryonic limb chondrogenesis and is associated with (cartilage) stemness (Oberlender and Tuan, 1994). Specific expression of NOTCH1 has been described at the developing cartilage surface of mouse knee joints (Hayes et al., 2003) and in cells with enhanced clonality at the articular surface of bovine cartilage (Dowthwaite et al., 2004). Therefore, we suggest that the specific combination and concentration of growth factors in PL as used in the current study drives chondrocytes into a dedifferentiated state with increased chondroprogenitor-like potential.

Next, our data demonstrated that chondrocytes that were expanded with PL maintain their chondrogenic differentiation capacities when subsequently cultured in a 3D environment. The deposition of GAGs was slightly increased in PL-expanded chondrocyte pellets, while type II collagen deposition was unaltered. Moreover, type I collagen production was absent in both FBS-expanded chondrocyte pellets as well as in PL-expanded chondrocyte pellets. This supports our suggestion that chondrocytes expanded in PL do dedifferentiate but maintain progenitor-like chondrogenic potential. Several previous studies also reported on an increase (Choi et al., 1980; Hildner et al., 2015) or maintenance (Pereira et al., 2013) of chondrogenic potential of chondrocytes after expansion in PL, while another study observed opposite outcomes (Sykes et al., 2018). This makes it hard to draw a conclusive statement on the effects of PL on chondrogenic capacities of chondrocytes is still lacking.

The effect of intra-articular PRP injections in the treatment of OA has been evaluated in various randomized controlled trials (Kon et al., 2011; Cerza et al., 2012; Patel et al., 2013; Filardo et al., 2015a; Smith, 2015; Cole et al., 2017; Jubert et al., 2017). Since some of these studies found a positive effect on pain and function (Kon et al., 2011; Cerza et al., 2012; Cole et al., 2017), we hypothesized that PL would have a stimulatory effect on chondrogenesis in pellets consisting of OA chondrocytes that are expanded in FBS-supplemented expansion medium. Interestingly, our results showed that culture in the presence of PL remarkably inhibited GAG deposition in the pellets when compared to control conditions and complete absence of type II collagen production was observed. Furthermore, real-time PCR data revealed a strong decrease in COL2A1 expression, indicating a dedifferentiation of the chondrocyte phenotype in presence of PL, even in 3D. To the best of our knowledge, no other studies have been performed in which the direct effect of PL on OA chondrocytes in 3D pellet culture is assessed. One paper reported on the effect of PL supplementation to the differentiation medium during only the first 3 days of chondrocyte pellet culture. However, these chondrocytes were also expanded using PL. These pellets performed significantly worse in cartilage matrix production when compared to pellets cultured in regular differentiation medium (Hildner et al., 2015). Outcomes of studies focusing on other 3D culture models are inconsistent. When chondrocytes were cultured in alginate beads and subjected to platelet supernatant, a product similar to PL, a distinct decrease in chondrogenic genes was measured when compared to control conditions lacking platelet supernatant (Gaissmaier et al., 2005). Others reported on induction of chondrogenesis when adding PRP or PL to a 3D culture system. When PL was incorporated in a dextran-containing hydrogel, it was able to enhance proliferation, and simultaneously, induce chondrogenesis, both for MSCs and for cocultures of MSCs and chondrocytes (Moreira Teixeira et al., 2012). The different findings might be explained by differences in the growth factor concentrations and ratios between the various growth factors.

The concentrated growth factors present in PL stimulate chondrocytes to proliferate. It is known that FGF and PDGF stimulate proliferation (Green et al., 2015; Zhao et al., 2016), while TGF-β inhibits proliferation and mediates initiation of differentiation in chondrocytes (Green et al., 2015). Furthermore, the cocktail of growth factors in the current study seems to maintain the cells in a progenitor-like state during expansion, demonstrated by enhanced expression of NOTCH1 and CDH2. These markers are involved in pathways that have been described to be of importance in chondrocyte proliferation during development. Proliferation and regeneration of chondrocytes *in vitro* are two distinct processes that do not go hand in hand. Upon expansion, chondrocytes present a dedifferentiated phenotype, with increased type I collagen gene expression (Darling and Athanasiou, 2005). However, the cells can recover their phenotype and redifferentiate when brought back into 3D culture (Marijnissen et al., 2000). Further in-depth characterization of PL-treated chondrocytes will be necessary to elucidate their phenotypical change in relation to chondroprogenitor cells and MSCs.

The same cocktail of growth factors seems to be ineffective in stimulating chondrocytes to produce cartilage matrix in a 3D pellet environment. Possibly the high concentrations of FGF in PRP may interfere with the redifferentiation process by suppressing cellular senescence and inhibition of the TGF-β pathway (Ito et al., 2007). Human platelets are a rich source of TGF-β and PRP can contain up to 100-fold increased concentrations of TGF-β (Eppley et al., 2004; Sundman et al., 2011). Accordingly, this study also found high concentrations of TGF-β in the used PL. It is known that overstimulation by TGF-β inhibits FGF- and Wnt-mediated proliferation of chondrocytes (Cleary et al., 2015). Besides that, it is likely that high concentrations of TGF-β play a role in *in vitro* fibrocartilage formation by chondrocytes (Narcisi et al., 2012). Future studies looking into molecular mechanisms causing chondrocytes to dedifferentiate upon PL-exposure can shed more light on the observations in the study presented here.

The variations in outcomes between this study and previous research can partly be explained by the numerous methods available to prepare PRP. Firstly, there is a great variation in platelet and growth factor concentration that is used in PRP, caused by both donor variability, as well as by the variety of preparation methods (Sundman et al., 2011; Mazzocca et al., 2012; Dhurat and Sukesh, 2014). Secondly, it remains unclear whether the concentration of growth factors in PRP is related to the concentration of platelets (Eppley et al., 2004; Sundman et al., 2011). Thirdly, there is a difference between leukocyte-rich (LR-) and leukocyte-poor (LP-)PRP. It is generally assumed that leukocytes and immune modulating cytokines present in PRP contribute to a pro-inflammatory environment and is undesirable in the treatment of OA (Anitua et al., 2015; Riboh et al., 2016; Simental-Mendía et al., 2018). Nonetheless, direct comparison in a clinical study between LP- and LR-PRP injections has not given conclusive outcomes which type of PRP is most effective in the treatment of OA (Filardo et al., 2012). However, an *ex vivo* study examining the effects of different PRP preparations on cartilage and meniscal explants suggested a pro-inflammatory effect of LR-PRP preparations (Kisiday et al., 2012). Clinical studies mainly use LP-PRP for injection, containing either similar numbers of leukocytes compared to whole blood (Kon et al., 2011; Filardo et al., 2015a), reduced amounts (Cerza et al., 2012; Cole et al., 2017; Jubert et al., 2017), or are deprived of leukocytes (Patel et al., 2013). There is a need for additional clinical studies to make clear which type of PRP is effective in the treatment of OA, and for PRP and PL preparation methods to be aligned between different laboratories, clinics, and hospitals (Fice et al., 2019). Objective measurements of cartilage quality after PRP treatment are not available to date. Comprehensive studies including objective analysis of cartilage quality after PRP injection are needed in addition to current analysis using mainly patient questionnaire outcomes. Furthermore, there are some limitations to *in vitro* models to consider. Laboratory models are usually limited to a single tissue or cell type, whereas PRP *in vivo* affects the whole joint environment (Filardo et al., 2015b). Moreover, positive clinical outcomes may be explained by mechanisms other than a direct effect of PL on the cells in the cartilage. It has been previously shown that PRP can act anti-inflammatory both *in vitro* as well as *in vivo* (Liu et al., 2014; Moussa et al., 2017). The inflammatory mediators in PRP and PL might create an improvement in the intra-articular environment, upon which cartilage matrix production by the resident chondrocytes is stimulated. An *in vitro* model as presented in the current study is unable to take these additional factors into account. More extensive studies using multiple cell types or tissues, or animal models are necessary to answer this question. Finally, *in vitro* culture does not mimic growth factor clearance, unless a bioreactor system is used. These factors should be taken into account when comparing *in vitro* results to clinical outcomes.

## Conclusion

The current study provides insights into the effects of PL on chondrogenic differentiation of human chondrocytes and its potential to be used for two different clinical applications. We propose that platelet lysate has potential to be used in the expansion of chondrocytes for application in cartilage defects. Nonetheless, PL exerts a strong inhibitory effect on chondrogenesis *in vitro*, suggesting caution for its utilization in intra-articular injections.

## Data Availability Statement

All datasets generated for this study are included in the article/Supplementary Material.

## Author Contributions

MR and LV: conception and design. MR: collection and assembly of data. MR, RL, and LV: analysis and interpretation of the data. MR, RL, JM, and LV: drafting of the article and reviewing, final approval of the article. All authors contributed to the article and approved the submitted version.

## Conflict of Interest

The authors declare that the research was conducted in the absence of any commercial or financial relationships that could be construed as a potential conflict of interest.

## Supplementary Material

FIGURE S1 Photomicrographs of control conditions of chondrocyte expansion. Chondrocytes expanded for 7 days with serum-free medium (negative control), medium containing 10% FBS (positive control), and experimental medium containing 5% platelet lysate (platelet lysate). Scale bars are 200 μm.

FIGURE S2 Histological evaluation of pellets consisting of platelet lysate-expanded chondrocytes. Safranin O staining **(top panel)** visualizing glycosaminoglycans (GAG) and type II collagen deposition visualized by immunohistochemistry **(bottom panel)** of all donors used in the experiment. Scale bars in the top panel are 100 μm. Scale bars in the bottom panel are 400 μm.

FIGURE S3 Histological evaluation of platelet lysate-treated pellets. Safranin O staining **(top panel)** visualizing glycosaminoglycans (GAG) and type II collagen deposition visualized by immunohistochemistry **(bottom panel)** of all donors used in the experiment. All scale bars are 100 μm.
