# Supplementary figures and images for: Importance of Timing of Platelet Lysate-Supplementation in Expanding or Redifferentiating Human Chondrocytes for Chondrogenesis
Source: Front Bioeng Biotechnol. 2020 Jul 8;8:804. doi: 10.3389/fbioe.2020.00804 (PMC7360809; doi:10.3389/fbioe.2020.00804)

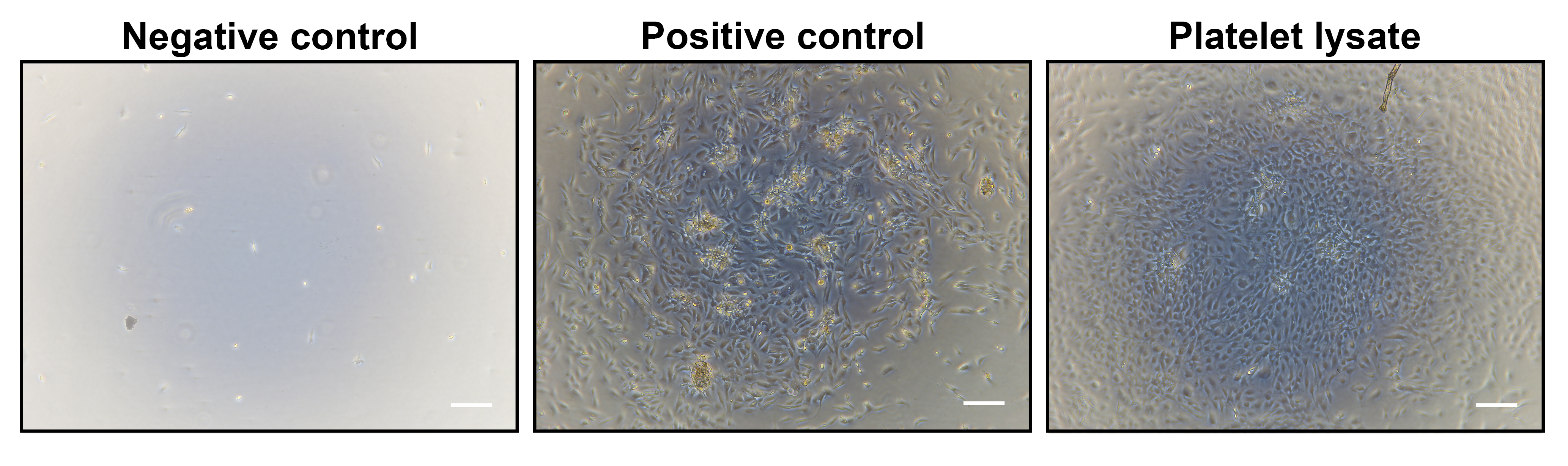

Supplement: FIGURE S1 — Photomicrographs of control conditions of chondrocyte expansion. Chondrocytes expanded for 7 days with serum-free medium (negative control), medium containing 10% FBS (positive control), and experimental medium containing 5% platelet lysate (platelet lysate). Scale bars are 200 μm. [file Image_1.tif]

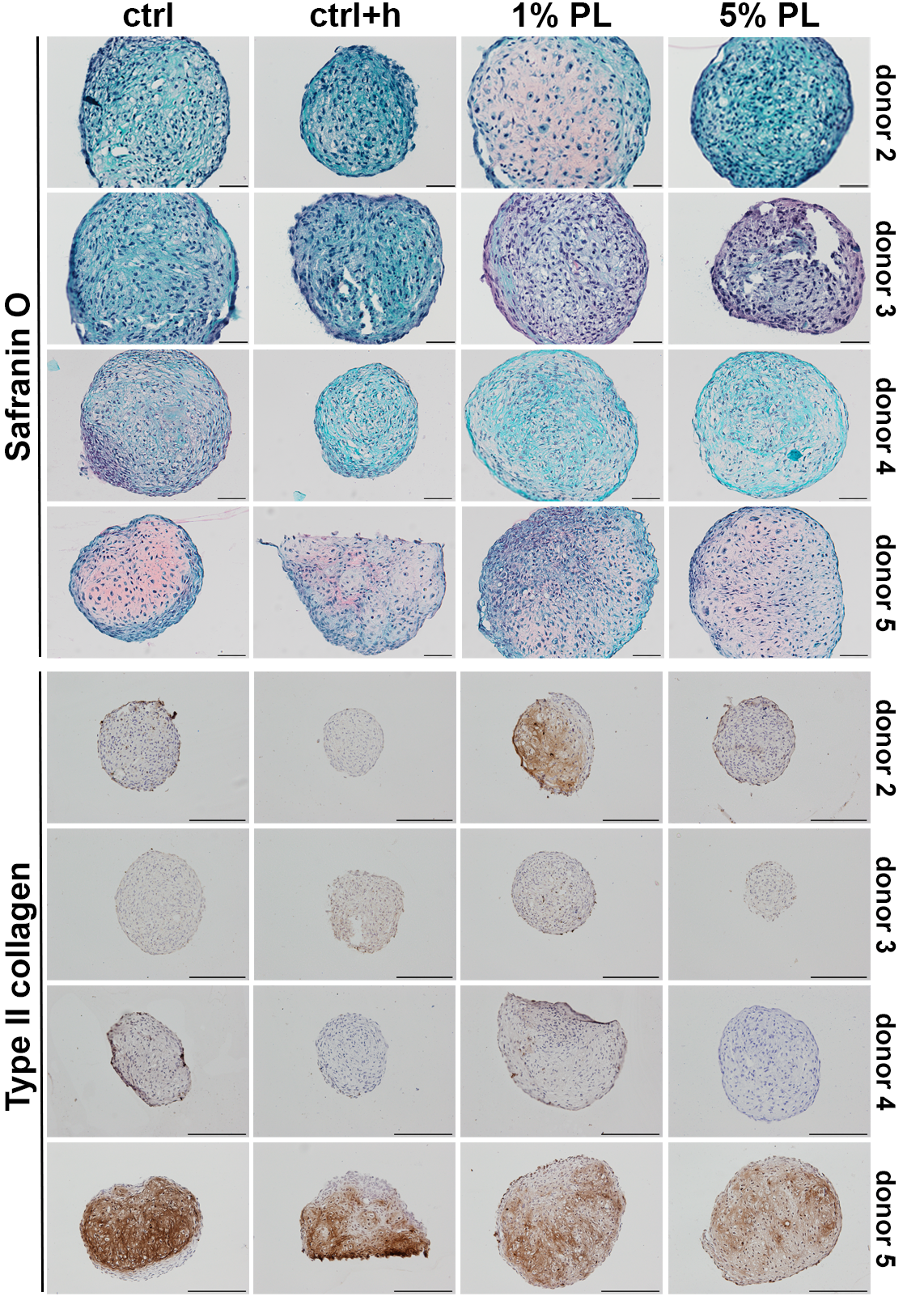

Supplement: FIGURE S2 — Histological evaluation of pellets consisting of platelet lysate-expanded chondrocytes. Safranin O staining (top panel) visualizing glycosaminoglycans (GAG) and type II collagen deposition visualized by immunohistochemistry (bottom panel) of all donors used in the experiment. Scale bars in the top panel are 100 μm. Scale bars in the bottom panel are 400 μm. [file Image_2.tif]

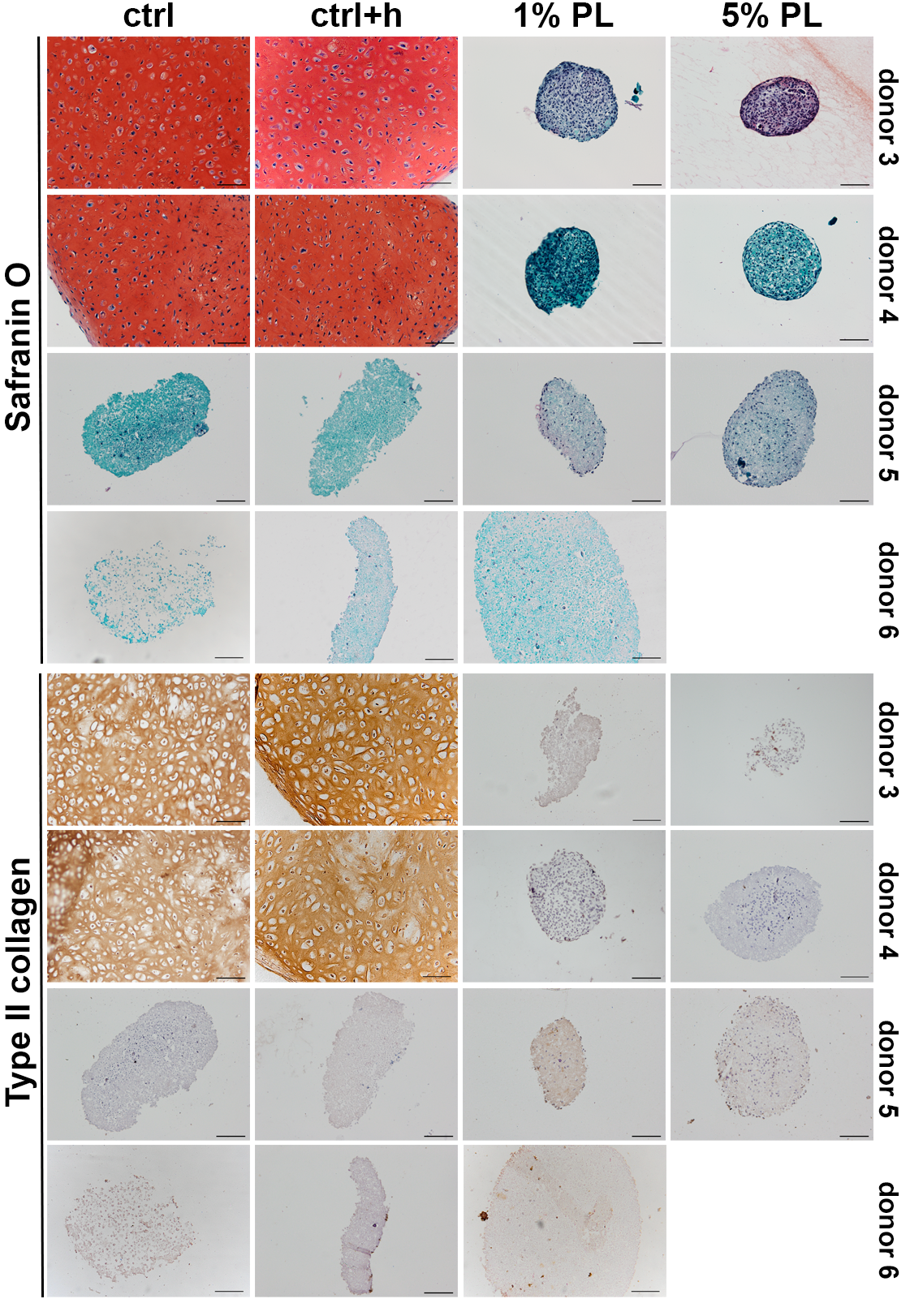

Supplement: FIGURE S3 — Histological evaluation of platelet lysate-treated pellets. Safranin O staining (top panel) visualizing glycosaminoglycans (GAG) and type II collagen deposition visualized by immunohistochemistry (bottom panel) of all donors used in the experiment. All scale bars are 100 μm. [file Image_3.tif]
